# Immune reconstitution syndrome-mediated acute fulminant tuberculous myopericarditis in young postpartum patient: a case report

**DOI:** 10.1093/ehjcr/ytaf542

**Published:** 2025-10-22

**Authors:** Mochamad Rizky Hendiperdana, Astrid Putri, Dyan Tony Cahya, Nugroho Sigit Hartanto

**Affiliations:** Division of Cardiovascular Medicine, Pandan Arang General Hospital, Kantil Road No. 14, Boyolali, Central Java 57316, Indonesia; Division of Cardiovascular Medicine, Pandan Arang General Hospital, Kantil Road No. 14, Boyolali, Central Java 57316, Indonesia; Division of Pulmonary Medicine, Pandan Arang General Hospital, Kantil Road No. 14, Boyolali, Central Java 57316, Indonesia; Department of Radiology, Pandan Arang General Hospital, Kantil Road No. 14, Boyolali, Central Java 57316, Indonesia

**Keywords:** Tuberculosis, Myopericarditis, Heart failure, Immune reconstitution syndrome, Case report

## Abstract

**Background:**

Postpartum symptom deterioration of tuberculosis is associated with the condition that is termed immune reconstitution syndrome (IRS). In the postpartum period, restitution of cellular immunity mediates the hypersensitivity reaction to *Mycobacterium tuberculosis* (MTB) antigen that hypothetically results in acute fulminant tuberculosis myopericarditis.

**Case summary:**

A 29-year-old woman develop acute respiratory distress 5 days following caesarean section. On admission vital sign indicating haemodynamic instability. Chest X-ray showed extensive pulmonary consolidation which signified for pulmonary tuberculosis. Echocardiography finding revealed left ventricular (LV) dysfunction with minimal pericardial effusion. Cardiac magnetic resonance imaging showed non-ischaemic late gadolinium enhancement (LGE) distribution. Upon to this finding, acute fulminant tuberculosis myopericarditis diagnosis was established. The patient then stabilized in intensive cardiac care unit with inotropic support, heart failure therapy, steroid, and anti-tuberculosis treatment (ATT). Patient clinical course was favourable and patient discharged at 8th admission day. At 3-month post-discharge visit, the patient showed complete LV recovery and chest X-ray also showed improvement in pulmonary consolidation.

**Discussion:**

Important to consider acute fulminant tuberculous myopericarditis that developed immediately in the postpartum period. The IRS-induced acute fulminant tuberculous myopericarditis is reasonable to be considered as an underlying mechanism. Most of the reported cases showed significant improvement after ATT initiation. Clinical vigilance and awareness for early diagnosis and delivering timely management of ATT as effective therapy for tuberculous myopericarditis is of the utmost importance.

Learning points

- Postpartum haemodynamic deterioration of TB is often associated with immune reconstitution syndrome (IRS). The IRS-induced fulminant TB myopericarditis must be enlisted as an underlying mechanism of postpartum acute haemodynamic instability.
- Early diagnosis and timely management of anti-tuberculosis therapy is of the utmost importance in the management of this reversible heart failure aetiology.

## Introduction

Tuberculosis is a global health burden particularly in the low-middle income countries that caused up to 1.4 million annual deaths worldwide.^1–5^ Postpartum symptom deterioration of tuberculosis is associated with the condition that is termed immune reconstitution syndrome (IRS).^6,7^ In the postpartum period, restitution of cellular immunity mediates the hypersensitivity reaction to *Mycobacterium tuberculosis* (MTB) antigen that hypothetically results in acute fulminant tuberculous myopericarditis.^7^ Herein, we reported a case of IRS-mediated postpartum fulminant tuberculous myopericarditis with left ventricular (LV) dysfunction which was successfully treated with anti-tuberculosis treatment (ATT), cortisteroid, and heart failure medication.

## Summary figure

**Table:** 

Timeline	Events
10 days prior to admission	Caesarean section procedure for the third pregnancy
5 days prior to admission	Severe breathlessness commenced with progressive worsening
Admission (Day 0)	Presented at emergency ward with unstable haemodynamic: diaphoretic and distressed with a heart rate 153 beats/minute and marked hypotension with blood pressure (BP) 65/31 (45) mmHg, peripheral oxygen saturation 95%, and respiratory rate 22 breaths/minute. Electrocardiography revealed marked sinus tachycardia with poor R wave progression. Chest X-ray showed cardiomegaly with diffuse pulmonary consolidation which signified for pulmonary tuberculosis. Laboratory results were increased in serum creatinine, lymphopenia, increased erythrocyte sedimentation rate, significant increase D-dimer and lactatemia (4.4 mmol/l). Echocardiography revealed global hypokinetic with LVEF of 34% with minimal pericardial effusion.
Day 1	Admitted to the intensive care unit for further management and haemodynamic stabilization.
Day 2	GDMT for heart failure start to be administered: ramipril 2.5 mg o.d., spironolactone 25 mg o.d., warfarin 2 mg o.d.
Day 3	Bisoprolol 2.5 mg o.d. was added. ATT with RHZE regimen: rifampicin 300 mg, isoniazid 150 mg, pyrazinamide 800 mg, and ethambutol 550 mg, and Intravenous hydrocortisone 100 mg/8 h was administered.
Day 5	CMR imaging showed LGE with midwall distribution at inferoseptal and inferolateral, subepicardial LGE at anteroseptal and anterolateral LV territory which signifies for non-ischaemic LGE distribution.
Day 8	Discharged uneventfully with serum lactate 1.5 mmol/l. Discharged medication: furosemide 40 mg o.d., ramipril 10 mg o.d., spironolactone 50 mg o.d., bisoprolol 5 mg o.d., hydrocortisone 20 mg o.d., and ATT.
One-month follow-up	Symptom improved with echocardiographic signs of LV function recovery with LVEF of 65%.
Three-month follow-up	Complete LV recovery with LVEF 61%. Chest X-ray showed improvement in pulmonary consolidation.

LVEF, left ventricular ejection fraction; GDMT, guideline-directed medical therapy; ATT, anti-tuberculosis treatment; CMR, cardiac magnetic resonance; LGE, late gadolinium enhancement.

## Case summary

A 29-year-old woman presented to the emergency department with severe breathlessness in 5 days after the caesarean section procedure for her third delivery. Dyspnoea worsened progressively until the patient presented at our centre. On admission vital signs: the patient looks diaphoretic and distressed with a heart rate 153 beats/minute and marked hypotension with blood pressure (BP) 65/31 (45) mmHg, peripheral oxygen saturation 95%, and respiratory rate 22 breaths/minute. The significant physical examination finding was bilateral lung crackles on chest auscultation. There was no history of pulmonary infection or any cardiac disease during recent and previous pregnancy. The patient noticed significant body weight reduction that was accompanied by non-productive cough 4 months prior to delivery.

**Figure 1:**
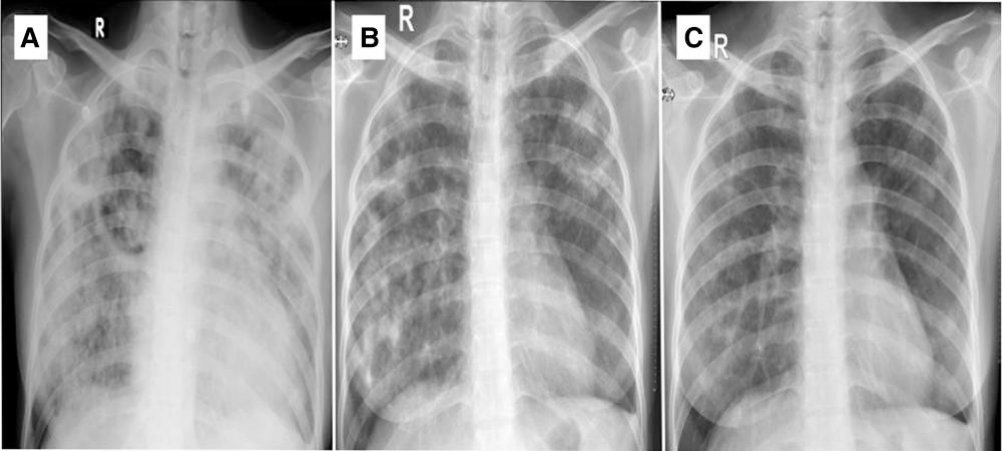
Serial chest roentgenography at (*A*) baseline initial admission; (*B*) 1-month post-hospital discharge; (*C*) 3 months after hospital discharge. The baseline X-ray showed diffuse consolidation over both lung lobes that followed gradual improvement of pulmonary consolidation at 1 month and 3 months after hospital discharge.

Electrocardiography revealed marked sinus tachycardia with poor R wave progression. Chest X-ray showed cardiomegaly with diffuse pulmonary consolidation which signified for pulmonary tuberculosis (*Figure 1A*). Laboratory results were euthyroid, increased in serum creatinine (2.8 mg/dl and eGFR 21.23 ml/min), lymphopenia 570 (n.v. 800–4000/µl), increased erythrocyte sedimentation rate to 54 mm/h, significant increase D-dimer >10 000 ng/ml and lactatemia (4.4 mmol/l). Other laboratory results were unremarkable.

**Figure 2:**
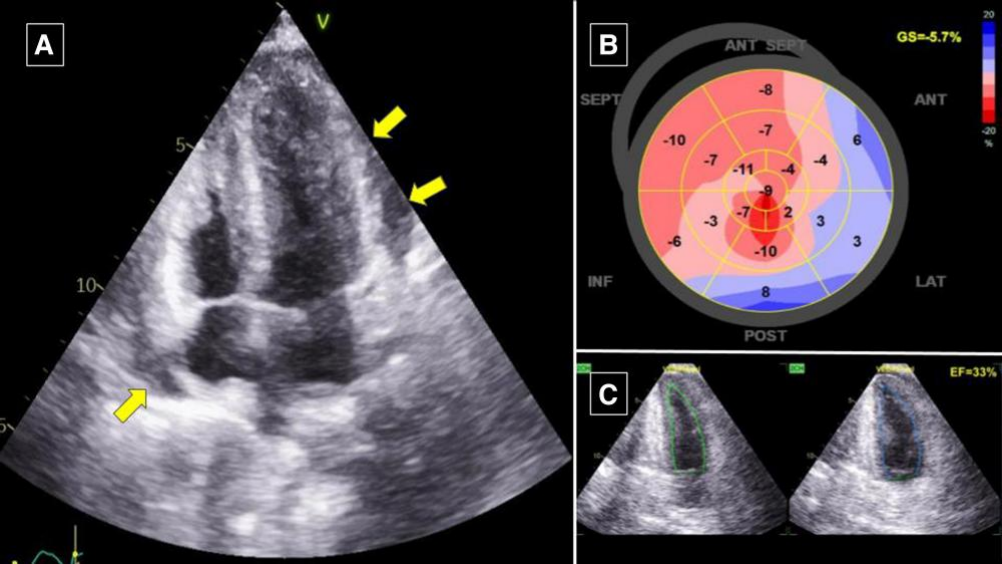
Baseline transthoracic echocardiography revealed (*A*) minimal pericardial effusion around the heart from apical 4-chamber view (arrow); (*B*) LV GLS (−5.7%); (*C*) global hypokinetic with LVEF of 34%. LV, left ventricular; GLS, global longitudinal strain; LVEF, left ventricular ejection fraction.

Transthoracic echocardiography revealed global hypokinetic LV with ejection fraction (LVEF) of 34%, LV GLS (−5.7%), minimal pericardial effusion was also observed, and other findings were insignificant (*Figure 2*) (see Supplementary material online, *Video S1*). Upon to these findings, the patient was diagnosed with acute heart failure and cardiogenic shock that suspiciously caused by two most possible aetiology. Firstly, it was highly suspected that the aetiology is peripartum cardiomyopathy (PPCM) and lastly, the tuberculous myopericarditis process.

The patient was admitted to the intensive cardiac care unit for further management and haemodynamic stabilization. The patient was administered dobutamine along with intravenous furosemide for decongestion purpose. Guideline-directed medical therapy (GDMT) for heart failure was administered with regimen ramipril 2.5 mg o.d., spironolactone 25 mg o.d., and warfarin 2 mg o.d. After several days of haemodynamic stabilization, bisoprolol 2.5 mg o.d. was added. Due to diagnosis establishment for a tuberculous process, the ATT with RHZE regimen (rifampicin 300 mg, isoniazid 150 mg, pyrazinamide 800 mg, and ethambutol 550 mg) and intravenous hydrocortisone 100 mg/8 h were administered.

**Figure 3:**
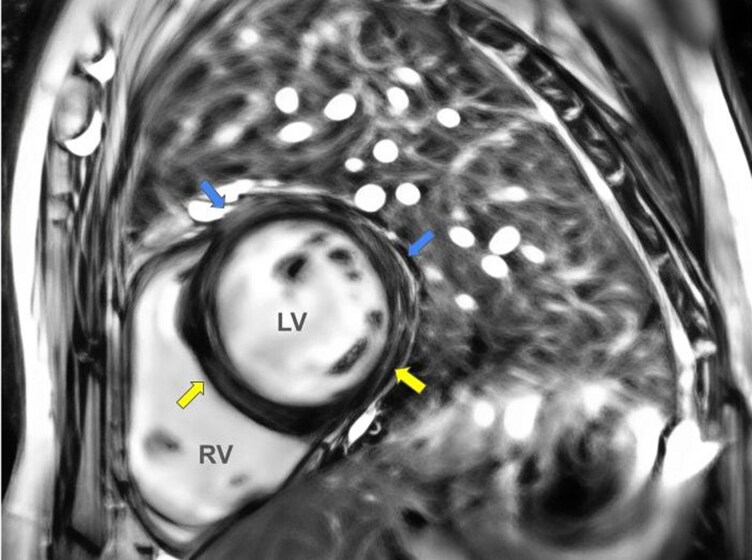
CMR imaging showed LGE with midwall distribution at inferoseptal and inferolateral, subepicardial LGE at anteroseptal and anterolateral LV territory which signifies for non-ischaemic LGE distribution. CMR, cardiac magnetic resonance; LGE, late gadolinium enhancement; LV, left ventricular.

At the 5th admission day, patient symptoms were resolved and haemodynamic was improved (HR 110 b.p.m., BP 100/50 mmHg). Serum lactate and creatinine were also improved (2.7 mmol/l and 1.2 mg/dl, respectively). Patient was then transferred to the medical ward with continuous GDMT uptitration. Cardiac magnetic resonance (CMR) imaging showed late gadolinium enhancement (LGE) with midwall distribution at inferoseptal and inferolateral, subepicardial LGE at anteroseptal and anterolateral LV territory which signifies for non-ischaemic LGE distribution (*Figure 3*). Patient then diagnosed as tuberculous myopericarditis.

At the 8th admission day, the patient was discharged uneventfully with stable haemodynamic and serum lactate 1.5 mmol/l with lactate clearance at the end of admission was 65.9%. The patient was discharged with medication furosemide 40 mg o.d., ramipril 10 mg o.d., spironolactone 50 mg o.d., bisoprolol 5 mg o.d., hydrocortisone 20 mg o.d., and ATT.

**Figure 4:**
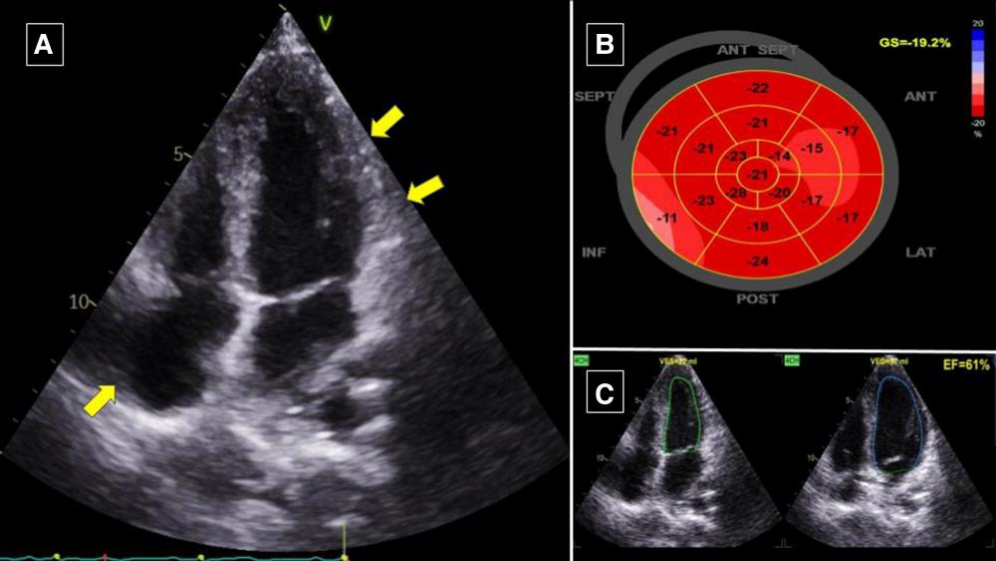
Three-month follow-up transthoracic echocardiography showed (*A*) resolution of pericardial effusion from apical four-chamber view (arrow); (*B*) significant improvement of LV GLS to (−19.2%); (*C*) significant improvement in LVEF of 61%. LV, left ventricular; GLS, global longitudinal strain; LVEF, left ventricular ejection fraction.

On an outpatient visit 1 month after discharge, the symptom resolved with echocardiographic signs of LV function recovery with LVEF of 65% with LV GLS (−15.3%). While symptom improvement was observed, we found significant change in chest X-ray infiltrate which depicted diffuse pulmonary tuberculosis (*Figure 1B*). Three months post-discharge visit, the patient showed complete LV recovery with LVEF 61% and LV GLS (−19.2%) (*Figure 4*). Chest X-ray also showed resolution of pulmonary consolidation (*Figure 1C*). The GDMT and ATT regimens were continued.

## Discussion

Myocardial involvement in tuberculosis is a rare case in published reports. The prevalence is 0.14%–2% from various series.^3,8,9^ The clinical presentation consists of ventricular arrhythmia, acute heart failure due to LV systolic dysfunction, and sudden cardiac death.^9^ This case was mimicking PPCM due to history of recent postpartum episodes. The presence of pericardial effusion in our case depicts pericardial involvement process. Hence, the aetiology of tuberculosis is more likely because of the myocardial involvement in tuberculosis is commonly in the form of myopericarditis.^8^

It is hypothesized that the immunological system adaptation that developed immediately in the postpartum period mediates the tuberculous myopericarditis by hypersensitivity reaction. Therefore, IRS phenomena possibly induced worsened tuberculosis in our patient which manifested as myopericarditis and acute heart failure.^6^ The possible explanation concerning the pathogenesis in this case was the involvement of immune-mediated hypersensitivity from IRS to the myocardium and pericardium. The clinicians had to put a high suspicion index of IRS in postpartum patients with unstable haemodynamic.

**Figure 5:**
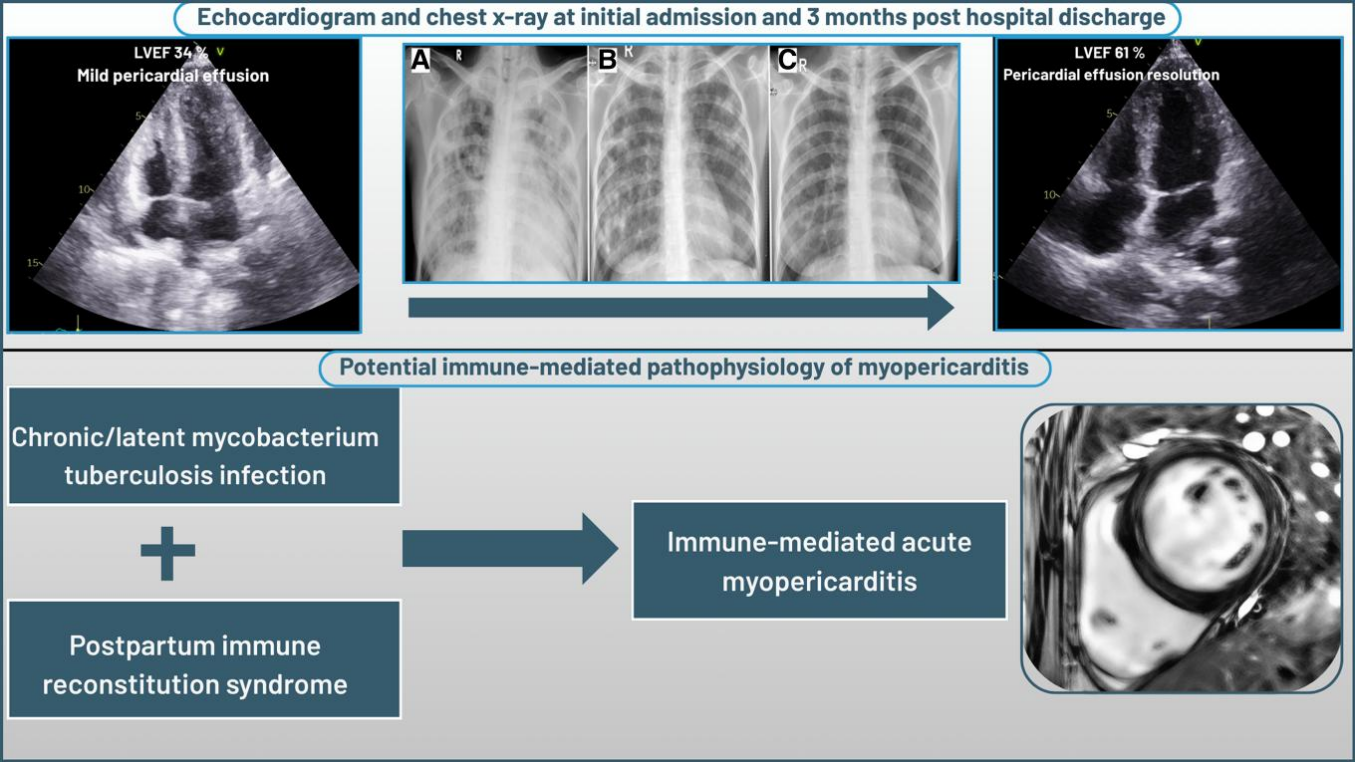
Visual summary of patient’s clinical course and proposed pathophysiological process of disease.

It is also important to consider the acute fulminant tuberculous myopericarditis in unexplained causes of heart failure that developed immediately in the postpartum period. The chronic and latent nature of tuberculous made recent infection is less likely. Therefore, the IRS-induced tuberculous myopericarditis is reasonable to be considered as an underlying mechanism (*Figure 5*).^6^ Unfortunately, endomyocardial biopsy for diagnosis establishment was not performed in our case due to resource limitation. Therefore, in our case, the administration of ATT was relied on TB suspicion from the chest X-ray that demonstrated diffuse pulmonary consolidation and CMR which showed non-ischaemic LGE distribution.

Raffali *et al.* reported a young woman with heart failure and disseminated TB who was successfully treated with ATT regimen and GDMT.^9^ On the other hand, Agarwal *et al.* and Cowley *et al.* reported a fatal case of acute fulminant tuberculous myocarditis that was later confirmed by post-mortem biopsy.^4,10^ Based on collected reports from Michira *et al.*, 81% of tuberculous myocarditis is predominant in young patients (below 45 years).^2^ While Shaikh *et al.* reported a similar case in an older man patient, which showed age distribution variability.^3^ Myocardial involvement of tuberculosis in the paediatric population has also been reported.^11,12^

Eighty per cent of tuberculous myocarditis fatal cases occurred in female patients with LV dysfunction whom also observed in our case.^2^ Fortunately, our case showed significant clinical response to ATT and GDMT with significant LV systolic function improvement several weeks after therapy. Most of the reported cases of tuberculous myopericarditis also showed significant improvement after ATT initiation.^5,9^ Unfortunately, due to the low incidence rate, subtle and slowly progressing nature of this condition made the antemortem diagnosis rarely.^2,10^ Therefore, clinician vigilance and awareness for early diagnosis and timely management of ATT and GDMT as effective therapy for tuberculous myopericarditis are of the utmost importance in the management of this reversible aetiology of heart failure,^1,2,4^ as demonstrated in our case that clinical improvement and myocardial reverse remodelling were observed. Since the immune-mediated hypersensitivity mechanism is prominent in this case, the role of systemic steroids to suppress immune response is also important to be considered as a myocarditis management although controversial.^7^

## Lead author biography


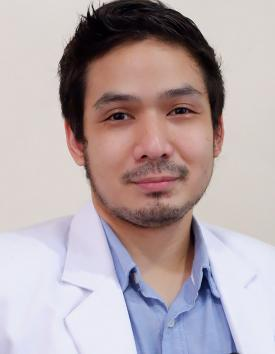


M. Rizky Hendiperdana MD, FIHA, currently a provider cardiology service at Pandan Arang General Public Hospital, Boyolali, Indonesia. He completed his training in cardiovascular medicine field at the Department of Cardiology and Vascular Medicine, Faculty of Medicine University of Indonesia/National Cardiovascular Centre Harapan Kita, Jakarta, Indonesia.

## Supplementary Material

ytaf542_Supplementary_Data

## Data Availability

The data underlying this article are available in the article and in its online supplementary materials.
